# Supplementary material for: HIV Pre-exposure Prophylaxis (PrEP): Knowledge, attitudes and counseling practices among physicians in Germany – A cross-sectional survey
Source: PLoS One. 2021 Apr 29;16(4):e0250895. doi: 10.1371/journal.pone.0250895 (PMC8084214; doi:10.1371/journal.pone.0250895)
Supplement: S1 File — Original German online version of the questionnaire used in the present survey. (PDF) [file pone.0250895.s001.pdf]

S1 File. Survey questionnaire (German version)

Sammons M, Gaskins M, Kutscha F, Nast A, Werner RN: HIV Pre-exposure Prophylaxis (PrEP): Knowledge, Attitudes and Counseling Practices among Physicians in Germany – A cross-sectional survey

Eine analoge Version des Fragebogens, die verwendet wurde, um die Kenntnisse und Einstellungen sowie die Beratungspraxis zur PrEP unter Berater*innen in öffentlichen und freigemeinnützigen HIV- und STI-Beratungsstellen zu erheben, wurde an anderer Stelle veröffentlicht: Kutscha F, Gaskins M, Sammons M, Nast A, Werner RN. HIV Pre-Exposure Prophylaxis (PrEP) Counseling in Germany: Knowledge, Attitudes and Practice in Non-governmental and in Public HIV and STI Testing and Counseling Centers. Front Public Health. 2020 Jul 14;8:298. doi: 10.3389/fpubh.2020.00298.

Einstellungen und Beratungspraxis zur HIV-Präexpositionsprophylaxe (PrEP)

unter Ärztinnen und Ärzten in Deutschland

Wir möchten Sie einladen, an einer anonymen Fragebogen-Studie zum Thema „Einstellungen und Beratungspraxis zur HIV-Präexpositionsprophylaxe (PrEP) unter Ärztinnen und Ärzten in Deutschland" teilzunehmen. Ziel der Studie ist zu ermitteln, was Ärztinnen und Ärzte in Deutschland zur PrEP denken und welche Probleme sich in der Beratung von Patienten ergeben. Die Studie soll Schwierigkeiten und Verbesserungspotenziale aufzeigen und so einen Beitrag zur Prävention von HIV-Infektionen leisten.

Die Studie besteht aus einem anonymen Fragebogen – es dauert etwa 10-15 Minuten, den Fragebogen auszufüllen.

Die Teilnahme ist freiwillig. Sie können Ihre Teilnahme jederzeit ohne Angabe von Gründen beenden.

Bitte nehmen Sie an dieser Umfrage nur teil, wenn Sie Arzt oder Ärztin sind. Bitte füllen Sie diesen Fragebogen nur einmal aus.

Worum geht es?

Die HIV-Präexpositionsprophylaxe (PrEP) ist eine seit August 2016 in Deutschland zugelassene Form der Prävention von Infektionen mit HIV. Durch die vorbeugende kontinuierliche oder Risikokontakt-bezogene Einnahme der Kombination zweier Wirkstoffe (Tenofovir disoproxil und Emtricitabin), besteht ein Schutz vor HIV-Infektion bei Sex auch ohne die Verwendung von Kondomen.

Die hohe Wirksamkeit und Sicherheit der PrEP konnte in verschiedenen randomisierten Studien und Kohortenstudien gezeigt werden. Die zunehmende Verbreitung der PrEP hat in einzelnen Großstädten (z.B. London, San Francisco) bereits zu einer Verringerung der Rate an HIV-Neuinfektionen geführt.

Auch in Deutschland spielt die PrEP als HIV-Präventionsstrategie eine zunehmende Rolle. Durch die mögliche Übernahme der Kosten für die Versorgung mit der PrEP durch die gesetzlichen Krankenkassen ist vor einer steigenden Anzahl an Beratungen zum Thema auszugehen.

Datenschutz

Wir garantieren Ihre Anonymität und werden keine Informationen über Sie erfassen, durch die Sie persönlich identifiziert werden können.

Die ausgefüllten anonymen Fragebögen werden an die Klinik für Dermatologie, Venerologie und Allergologie an der Charité – Universitätsmedizin Berlin übermittelt und dort statistisch ausgewertet. Ein Bericht der Umfrage wird voraussichtlich Ende 2019 erstellt und in einer wissenschaftlichen Zeitschrift publiziert werden. Möglicherweise werden weitere Berichte von den Umfrageergebnissen auf medizinischen Kongressen veröffentlicht.

Hier werden keine Daten veröffentlicht, die einen Rückschluss auf die einzelnen Teilnehmer ermöglichen.

Weitere Hinweise zum Datenschutz:

Im Rahmen unserer Umfrage erheben und verarbeiten wir Informationen über Ihre Person (Geschlecht, Alter, Fachgebiet, Weiterbildungsstatus) und über Ihr Arbeitsumfeld (erste drei Ziffern der Postleitzahl Ihres Arbeitsorts), ohne aber den Namen oder eindeutige Bezugsgrößen zu erheben, die einen unmittelbaren Bezug zu Ihrer Person ermöglichen.

Die Teilnahme an dieser Umfrage soll anonym erfolgen ohne die Nennung Ihres Namens oder Daten, die eine Identifikation ermöglichen. Dies bitten wir auch beim Ausfüllen des Fragebogens zu berücksichtigen, da eine spätere Korrektur nicht möglich sein wird.

Sollte jedoch im Einzelfall aufgrund der Beantwortung der Fragen ein Rückschluss auf Ihre Identität möglich sein, steht Ihnen das Fragerecht und das Beschwerderecht zur Verfügung.

Wir bitten Sie höflich, uns bei der Umsetzung des Gebotes der Datenminimierung zu unterstützen, indem Sie bei der Beantwortung der Fragen darauf achten, uns nur die abgefragten, für den konkreten Studienzweck erforderlichen personenspezifischen Angaben zu erteilen.

Im Falle von **Fragen oder Anmerkungen**, wenden Sie sich gerne an den verantwortlichen Studienleiter:
Dr. med. Ricardo N. Werner
Klinik für Dermatologie, Venerologie und Allergologie
Charité – Universitätsmedizin Berlin
Charitéplatz 1
10117 Berlin
debm01@charite.de

Bei **Anliegen zur Datenverarbeitung** und zur Einhaltung der datenschutzrechtlichen Anforderungen können Sie sich auch an die Stabsstelle Datenschutz der Charité wenden:
Stabsstelle Datenschutz
Charitéplatz 1
10117 Berlin
Telefon: 030 450580016
E-Mail: datenschutz@charite.de

*** Bitte kreuzen Sie an:**
O Ich habe die Studieninformation gelesen und möchte an dieser Umfrage teilnehmen.

<u>Zunächst einige Fragen zu Ihnen…</u>

In was für einer Einrichtung sind Sie tätig?
O In einer Praxis (Inhaber*in)
O In einer Praxis (angestellt)
O In einem Krankenhaus
O In einer Universitätsklinik
O Sonstiges: ___________

Welchem medizinischen Fachgebiet gehören Sie an?
(Mehrfachnennung möglich)
O Allgemeinmedizin
O Innere Medizin
O Urologie
O Haut- und Geschlechtskrankheiten
O Zusatzweiterbildung Infektiologie
O Sonstiges: ___________

Was ist Ihr Weiterbildungsstatus in Ihrem Fachgebiet?
O Facharzt/ärztin
O Assistenzarzt/ärztin

Bitte geben Sie die ersten drei Ziffern der Postleitzahl Ihrer Praxis / Ihres Arbeitsortes an: ____

Sind Sie in einer Praxis bzw. Klinik mit Schwerpunkt in der Versorgung von Menschen mit HIV tätig?
O Ja
O Nein

Wie alt sind Sie?
______ Jahre

Was ist Ihr Geschlecht?
Bitte wählen Sie nur eine der folgenden Antworten aus:
O Weiblich
O Männlich
O Divers

In welchen weiteren Sprachen können Sie Ihre Patientinnen und Patienten beraten?
(Mehrfachnennung möglich)
O Arabisch
O Dänisch
O Englisch
O Französisch
O Italienisch
O Niederländisch
O Polnisch
O Russisch
O Spanisch
O Tschechisch
O Türkisch
O Weitere: ______________

Einige Fragen zu Ihrem Praxisalltag...

Bitte schätzen Sie:
Wie viele Personen werden in Ihrer Praxis bzw. Klinik durchschnittlich in einem Quartal auf HIV getestet? ca. _______

Bitte schätzen Sie:
Bei wie vielen Personen stellen Sie durchschnittlich in einem Quartal die Diagnose einer HIV-Infektion? ca. _______

Bitte schätzen Sie:
Wie viele männliche Patienten, von denen Sie wissen, dass sie Sex mit Männern haben (MSM), und Transgender-Personen sehen Sie durchschnittlich in einem Quartal? ca. _______

... und zu Ihrer Berufspraxis

Den Empfehlungen der Deutsch-Österreichischen Leitlinien zur HIV-Präexpositionsprophylaxe* zufolge soll die PrEP unter anderem HIV-negativen MSM oder Transgender-Personen angeboten werden, wenn mindestens eines der folgenden Kriterien zutrifft:

- Angabe von analem Sex ohne Kondom innerhalb der letzten 3-6 Monate
- Angabe, voraussichtlich in den nächsten Monaten analen Sex ohne Kondom zu haben
- Diagnose einer sexuell übertragbaren Infektion (STI) in den letzten 12 Monaten

* Deutsch-Österreichische Leitlinien zur HIV-Präexpositionsprophylaxe, AWMF-Register-Nr.: 055-008, https://daignet.de/site-content/hiv-therapie/leitlinien-1

Die folgenden Fragen beziehen sich auf die oben genannte Leitlinie.

Bitte schätzen Sie, bezogen auf ein durchschnittliches Quartal:
Wie viele Personen aus der oben genannten, in der Leitlinie definierten Personengruppe...

...sehen Sie in Ihrer Praxis? ca. _______

...beraten Sie zum Thema PrEP? ca. _______

...sprechen von sich aus das Thema PrEP an? ca. _______

...werden von Ihnen auf das Thema PrEP angesprochen? ca. _______

...bekommen von Ihnen PrEP verordnet? ca. _______

<u>Einige Fragen zu Ihrer Praxis im Umgang mit PrEP</u>

Wenn eine Person aus den folgenden Personengruppen in Ihrer Sprechstunde ist, würden Sie sie auf das Thema PrEP aktiv ansprechen?
Bitte wählen Sie die zutreffende Antwort für jeden Punkt aus:

	Keinesfalls	*Wahrscheinlich nicht*	*Vielleicht*	*Ziemlich wahrscheinlich*	*Ganz sicher*
MSM oder Transgender-Personen, die Ihnen berichten, außerhalb einer monogamen Beziehung Analsex zu haben, ohne Kondome zu verwenden	O	O	O	O	O
MSM oder Transgender-Personen, die Ihnen berichten, Sex mit Gelegenheitspartnern zu haben	O	O	O	O	O
MSM oder Transgender-Personen, bei denen Sie erstmalig eine bakterielle STI (z.B. Syphillis, Gonorrhoe, Chlamydien) diagnostizieren	O	O	O	O	O
MSM oder Transgender-Personen, bei denen Sie wiederholt bakterielle STIs (z.B. Syphillis, Gonorrhoe, Chlamydien) diagnostizieren	O	O	O	O	O
MSM oder Transgender-Personen, bei denen Sie eine nicht-bakterielle STI (z.B. Herpes genitalis, Condylomata acuminata) diagnostizieren	O	O	O	O	O
MSM oder Transgender-Personen, die Ihnen berichten, eine HIV-Postexpositionsprophylaxe einzunehmen, eingenommen zu haben oder diese von Ihnen verordnet bekommen	O	O	O	O	O
MSM oder Transgender-Personen, die angeben, Sex unter Einfluss von Drogen zu haben ("Chemsex")	O	O	O	O	O

MSM oder Transgender-Personen in einer Beziehung mit einem HIV-positiven Partner, dessen Viruslast nicht unterhalb der Nachweisgrenze ist	O	O	O	O	O

Einige Fragen zu Ihren Kenntnissen und Einstellungen zur PrEP

Wie sehr stimmen Sie den folgenden Aussagen zu? *(Items in randomisierter Reihenfolge präsentiert)*

	Stimme gar nicht zu	*Stimme eher nicht zu*	*Teils-teils*	*Stimme eher zu*	*Stimme voll zu*
„Ich kenne mich gut mit PrEP aus"	O	O	O	O	O
„Ich kann Personen umfassend dazu beraten, ob in ihrem jeweiligen Fall die Einnahme von PrEP sinnvoll ist"	O	O	O	O	O
„Ich kann Personen zu den möglichen Nebenwirkungen der PrEP umfassend beraten"	O	O	O	O	O
„Ich kann Personen zu den möglichen Einnahmemodalitäten der PrEP (z.B. kontinuierlich oder Risikokontakt-bezogen) umfassend beraten"	O	O	O	O	O
„Ich kann Personen zu den erforderlichen medizinischen Begleituntersuchungen zur PrEP umfassend beraten"	O	O	O	O	O

Wie sehr stimmen Sie den folgenden Aussagen zu? *(Items in randomisierter Reihenfolge präsentiert)*

	Stimme gar nicht zu	*Stimme eher nicht zu*	*Teils-teils*	*Stimme eher zu*	*Stimme voll zu*
„PrEP ist ein wichtiger Bestandteil von Präventionsstrategien	O	O	O	O	O

gegen HIV“					
„PrEP ist eine verlässliche Methode, sich vor HIV zu schützen"	O	O	O	O	O
„PrEP ist eine nebenwirkungsarme Methode, sich vor HIV zu schützen“	O	O	O	O	O
„PrEP ist unnötig, denn es gibt bessere Alternativen, um sich vor HIV zu schützen"	O	O	O	O	O
„Die Versorgung mit PrEP sollte von der gesetzlichen Krankenkasse bezahlt werden“	O	O	O	O	O
„PrEP verbessert die Lebensqualität der Nutzer“	O	O	O	O	O
„Wenn die PrEP Personen mit hohem Risiko für eine HIV Infektion verordnet wird, ist das eine kosteneffektive Maßnahme“	O	O	O	O	O
„Es ist unethisch, gesunden Menschen eine tägliche Medikation zur Prävention von HIVInfektionen zu verschreiben“	O	O	O	O	O

Jetzt noch zu Ihren Erfahrungen mit der Verordnung der PrEP...

Wie oft ist Ihnen in den letzten 24 Monaten Folgendes passiert?

Sie haben einem Patienten empfohlen, mit PrEP zu beginnen, aber der Patient lehnte dies ab. Zu einem späteren Zeitpunkt ist der Patient HIV-serokonvertiert.
Ca. _________

Sie haben einem Patienten empfohlen, mit PrEP zu beginnen, aber der Patient wollte zunächst darüber nachdenken. Zu einem späteren Zeitpunkt ist der Patient HIV-serokonvertiert.
Ca. _________

Ein Patient bekundet Interesse, die PrEP einzunehmen, es stellt sich aber zum Zeitpunkt der Einleitungsuntersuchung heraus, dass der Patient bereits HIV-positiv ist.
Ca. _________

Wenn diese Ereignisse auftraten, was waren Ihrer Einschätzung nach die mutmaßlichen Gründe für die Ablehnung bzw. zu späte Entscheidung, PrEP zu nehmen? ______________________

Fast fertig. Jetzt noch zu möglichen Barrieren zur Verordnung und Einnahme von PrEP...

Welche Relevanz haben Ihrer persönlichen Erfahrung nach die folgenden Barrieren zur Verordnung und Einnahme der PrEP?

	Keine Relevanz – 0	*1*	*2*	*3*	*4*	*5*	*6*	*7*	*8*	*9*	*Höchste Relevanz – 10*
Patienten haben Sorgen vor leichten/vorübergehenden Nebenwirkungen	O	O	O	O	O	O	O	O	O	O	O
Patienten haben Sorgen vor schweren/bleibenden Nebenwirkungen	O	O	O	O	O	O	O	O	O	O	O
Patienten haben Sorgen, sich mit anderen sexuell übertragbaren Erkrankungen zu infizieren	O	O	O	O	O	O	O	O	O	O	O
Die monatlichen Kosten für die PrEP-Medikamente sind für Patienten zu hoch	O	O	O	O	O	O	O	O	O	O	O
Die Kosten für notwendige Folgeuntersuchungen (STI-Screening etc.) sind für Patienten zu hoch	O	O	O	O	O	O	O	O	O	O	O
Kulturelle Probleme halten Patienten von der Einnahme einer PrEP ab	O	O	O	O	O	O	O	O	O	O	O
Die Patienten schätzen ihr eigenes Risiko, sich mit HIV zu infizieren als zu gering ein	O	O	O	O	O	O	O	O	O	O	O
Der Zeitaufwand, regelmäßige Arztbesuche und/oder STI-Screenings wahrzunehmen, ist für Patienten zu hoch	O	O	O	O	O	O	O	O	O	O	O
Patienten haben Schwierigkeiten, einen Arzt/eine Ärztin zu finden, der/die ihnen PrEP verordnet	O	O	O	O	O	O	O	O	O	O	O
Die Patienten haben Sorgen vor sozialer Stigmatisierung in der peer group	O	O	O	O	O	O	O	O	O	O	O
Patienten haben zu wenig Informationen über PrEP in Patienten-gerechter Sprache	O	O	O	O	O	O	O	O	O	O	O
Patienten fehlen Informationen zur PrEP in ihrer Muttersprache	O	O	O	O	O	O	O	O	O	O	O
Für Ärztinnen und Ärzte ist das Management von PrEP-Patienten zu zeitaufwendig	O	O	O	O	O	O	O	O	O	O	O
Es ist schwierig für Ärztinnen und Ärzte, die Personen zu	O	O	O	O	O	O	O	O	O	O	O

identifizieren, die von einer PrEP profitieren würden											

Gibt es weitere relevante Probleme für potenzielle PrEP-Nutzer*innen, die in Ihrer persönlichen Beratungspraxis auftreten?
Diese können Sie uns hier gerne mitteilen... ____

__Zwei letzte Fragen:__

Welche der folgenden Informations- oder Schulungsmaterialien würden die Beratungen zur PrEP praktikabler machen oder verbessern? (Mehrfachnennung möglich)

O Eine Leitlinie mit übersichtlicher Darstellung von Indikationen, Kontraindikationen und erforderlichen Untersuchungen
O Eine Entscheidungshilfe für Patient*innen, die Informationen zur PrEP in Patient*innen-verständlicher Art darstellt
O Eine Entscheidungshilfe für Patient*innen, die Informationen zur PrEP in unterschiedlichen Sprachen darstellt
O Eine App- oder SMS-gestützte Erinnerungsfunktion für PrEP-User, um deren Adhärenz (regelmäßige Einnahme)zu fördern
O Informationen oder Schulungen für Ärztinnen und Ärzte zum Management von PrEP
O Informationen oder Schulungen für Ärztinnen und Ärzte zur Identifikation von Patient*innen, die von PrEP profitieren könnten
O Informationen oder Schulungen zum Thema „Mit Patient*innen über Sexualität sprechen“
O Sonstiges: _____________

Gibt es weitere Aspekte oder Probleme im Zusammenhang mit PrEP, die Sie beschäftigen? Haben Sie andere Vorschläge, wie die Beratung und Versorgung von Menschen, die von einer PrEP möglicherweise profitieren könnten, verbessert werden kann?
Diese können Sie uns hier gerne mitteilen... ____________________

Vielen Dank für Ihre Teilnahme an der Erhebung!
