# Supplementary material for: HIV Pre-exposure Prophylaxis (PrEP): Knowledge, attitudes and counseling practices among physicians in Germany – A cross-sectional survey
Source: PLoS One. 2021 Apr 29;16(4):e0250895. doi: 10.1371/journal.pone.0250895 (PMC8084214; doi:10.1371/journal.pone.0250895)
Supplement: S2 File — English translation of the online version of the questionnaire used in the present survey. Please note that the present translation has been undertaken for the publication only). (PDF) [file pone.0250895.s002.pdf]

S2 File. Survey questionnaire (English translation*)

Sammons M, Gaskins M, Kutscha F, Nast A, Werner RN: HIV Pre-exposure Prophylaxis (PrEP): Knowledge, Attitudes and Counseling Practices among Physicians in Germany – A cross-sectional survey

* Please note that the questionnaire was only available in German language for the survey and that the present translation has been undertaken for the publication only. A related version of the questionnaire which we developed to explore PrEP knowledge, attitudes and counselling practices among non-governmental counselling centres and local health offices in Germany has been published elsewhere: Kutscha F, Gaskins M, Sammons M, Nast A, Werner RN. HIV Pre-Exposure Prophylaxis (PrEP) Counseling in Germany: Knowledge, Attitudes and Practice in Non-governmental and in Public HIV and STI Testing and Counseling Centers. Front Public Health. 2020 Jul 14;8:298. doi: 10.3389/fpubh.2020.00298.

Attitudes and counselling practice regarding HIV pre-exposure prophylaxis (PrEP) among physicians in Germany

We would like to give you the opportunity to participate in an anonymous survey of the attitudes and counselling practice regarding HIV pre-exposure prophylaxis (PrEP). The aim of this study is to assess what physicians in Germany think about PrEP and what problems arise when counselling patients. The study aims at identifying difficulties, potential for improvement, and thus, at contributing to the prevention of HIV infections.

The survey is anonymous and takes approximately 10-15 minutes to complete.

Participation in the survey is voluntary. You can cancel your participation at any time without giving a reason.

Please only take part in this survey if you are a physician. Please complete the survey only once.

<u>What is the survey about?</u>

HIV pre-exposure prophylaxis (PrEP) is a form of prevention of infections with HIV that has been approved in Germany in August 2016. The continuous or on-demand intake of the combination of tenofovir disoproxil and emtricitabine provides protection against HIV infection during sex even without using condoms.

The high efficacy and safety of PrEP has been shown in various randomized controlled studies and cohort studies. The implementation of PrEP has already led to a decrease in the incidence of new HIV infections among men who have sex with men in some major cities (e.g. London, San Francisco).

PrEP also plays an increasing role in Germany. Due to the expected inclusion of PrEP in the benefits catalogue of the statutory health insurance, an increasing number of consultations on the topic is expected.

Data protection

We guarantee your anonymity. No information is collected that can be used to identify you personally.

The data of the completed anonymous questionnaire are transmitted to the Department of Dermatology, Venereology and Allergology at Charité - Universitätsmedizin Berlin, where the data will be evaluated and statistically analyzed. A report of the survey is expected to be prepared at the end of 2019 and published in a scientific journal. Possibly, further reports of the survey results will be published at medical congresses.

No data will be published that allow conclusions about the individual participants.

Further notes on data protection:

As part of our survey, we collect and process information about you (gender, age, specialty, continuing education status) and about your working context (first three digits of the zip code of your place of work), but without collecting your name or any clear reference data that would allow a direct link to your person.

Participation in this survey is to be anonymous without mentioning your name or any data that would allow identification. We also ask you to keep this in mind when filling out the questionnaire, as it will not be possible to correct it at a later date.

However, should it be possible to infer your identity in individual cases on the basis of the answers to the questions, you have the right to ask questions and the right to complain.

We kindly ask you to support us in implementing the principle of data minimization by making sure when answering the questions that you only provide the information requested and necessary for the specific purpose of the study.

In case of questions or comments, please contact the responsible study director:
Dr. med. Ricardo N. Werner
Klinik für Dermatologie, Venerologie und Allergologie
Charité – Universitätsmedizin Berlin
Charitéplatz 1
10117 Berlin
debm01@charite.de

If you have any concerns about data processing and compliance with data protection requirements, you can also contact the Charité Data Protection Office:

Stabsstelle Datenschutz
Charitéplatz 1
10117 Berlin
Telefon: 030 450580016
E-Mail: datenschutz@charite.de

*** If you want to participate in this survey, please confirm by checking the box:**

O I have read the study information and would like to take part in this survey.

Some questions about you first...

In what type of facility do you work?
O In an office-based practice (owner)
O In an office-based practice (employee)
O In a hospital
O In a university hospital
O Other: ___________

What is your medical specialty?
(Multiple answers allowed)
O General medicine (general practitioner)
O Internal medicine
O Urology
O Dermatology and Venereology
O Additional training in infectious diseases
O Other: ___________

What is your training status in your specialty?
O Senior physician (specialist)
O Junior physician (registrar)

Please provide the first three digits of your practice/work location zip code: ____

Do you work in a practice or clinic certified according to the German quality assurance agreement for HIV / PrEP care?
O Yes
O No

What is your age?
______ years

What is your gender?
O Female
O Male
O Diverse

In which further languages can you advise your patients?
(Multiple answers allowed)
O Arabic
O Danish
O English
O French
O Italian
O Dutch
O Polish
O Russian
O Spanish
O Czech
O Turkish
O Other: ______________

Some questions about your day-to-day practice....

Please estimate:
On average, how many persons get tested for HIV in your practice or clinic in a quarter year? Approx. _______

Please estimate:
On average, how many persons are diagnosed with HIV in a quarter year? approx. _______

Please estimate:
On average, how many male persons you know have sex with men (MSM) and trans persons do you see in a quarter year? approx. _______

…and about your PrEP counselling practice

According to the German-Austrian guidelines on HIV pre-exposure prophylaxis*, PrEP should be offered to HIV-negative MSM or transgender people who meet at least one of the following criteria:

- History of anal sex without a condom in the past 3-6 months
- Indication of likely having anal sex without a condom in the next months
- History of a sexually transmitted infection (STI) in the past 12 months

* German-Austrian guidelines on HIV pre-exposure prophylaxis, Deutsch-Österreichische Leitlinien zur HIV-Präexpositionsprophylaxe, AWMF-Register-Nr.: 055-008, https://daignet.de/site-content/hiv-therapie/leitlinien-1

The following three questions relate to appointments with persons who meet these criteria.

Please estimate, based on an average quarter year:
How many persons who meet the definition of MSM and trans persons who should be offered PrEP according to the guideline...

...do you see in your practice? Approx. _______

...do you counsel on the topic of PrEP? approx. _______

...do you address the topic of PrEP on your own initiative? ca. _______

...are approached by you on the subject of PrEP? ca. _______

...are prescribed PrEP by you? approx. _______

<u>Some questions about your practice in dealing with PrEP.</u>

If you see a person from one of the following groups of people in your consultation, how likely is it that you proactively give this person advice about PrEP?
Please select the applicable answer for each item:

	Not at all	*Probably not*	*Maybe*	*Quite likely*	*Quite sure*
MSM or trans persons who report not using condoms when having sex (outside a monogamous partnership)	O	O	O	O	O
MSM or trans persons who report having sex with occasional partners	O	O	O	O	O
MSM or trans persons in whom you diagnose a bacterial STI (e.g. Syphillis, gonorrhea, chlamydia) for the first time	O	O	O	O	O
MSM or trans persons in whom you diagnose a bacterial STI (e.g. Syphillis, gonorrhea, chlamydia) repeatedly	O	O	O	O	O
MSM or trans persons in whom you diagnose a non-bacterial STI (e.g. genital herpes, anogenital warts)	O	O	O	O	O
MSM or trans persons who report currently taking HIV postexposure prophylaxis or who have been prescribed PEP by you	O	O	O	O	O
MSM or trans personen who report using recreational drugs during sex ("chemsex")	O	O	O	O	O
MSM or trans persons with an HIV-positive partner, whose viral load is not undetectable	O	O	O	O	O

<u>Some questions about your knowledge and attitudes towards PrEP.</u>

Do you agree or disagree with the following statements? (Items presented in randomized order)

	Strongly disagree	*Disagree*	*Neither agree nor disagree*	*Agree*	*Strongly agree*
"I am well-informed about PrEP"	O	O	O	O	O
"I am able to comprehensively give patients advice on whether it makes sense to take PrEP in their respective case"	O	O	O	O	O
"I am able to comprehensively give patients advice on the adverse effects of PrEP"	O	O	O	O	O
"I am able to comprehensively give patients advice on the possible modalities of intake of PrEP (e.g., continuous vs. on-demand)"	O	O	O	O	O
"I am able to comprehensively give patients advice on the medical investigations necessary during the use of PrEP"	O	O	O	O	O

Do you agree or disagree with the following statements? (Items presented in randomized order)

	Strongly disagree	*Disagree*	*Neither agree nor disagree*	*Agree*	*Strongly agree*
"I think that PrEP is an important element of HIV prevention strategies"	O	O	O	O	O
"I think that PrEP is a reliable method to protect oneself from HIV"	O	O	O	O	O
"I think that PrEP is a method to protect oneself from HIV that has few side effects"	O	O	O	O	O
"I think that PrEP is unnecessary, because there are better alternatives to protect oneself from HIV"	O	O	O	O	O
"I think that PrEP should be paid for by the statutory health insurance"	O	O	O	O	O
“PrEP improves the user’s quality of life”	O	O	O	O	O
“If is prescribed to people at high risk for HIV infection, PrEP is cost-effective”	O	O	O	O	O
“It is unethical to prescribe daily medication to healthy people to prevent HIV infection”	O	O	O	O	O

<u>What are your experiences with prescribing PrEP....</u>

In the last 24 months, how often has the following happened to you?

You recommended a patient to start with PrEP, but the patient declined. At a later point in time, the patient acquired an infection with HIV.
Approximately _________

You recommended a patient to start with PrEP, but the patient wanted to think about it first. At a later point in time, the patient acquired an infection with HIV.
Approx. _________

A patient expressed interest in taking PrEP, but it turned out at the time of initiation that the patient was HIV-positive already.
Approx. _________

If any of these events occurred, what do you think were the presumed reasons for declining or deciding too late to start with PrEP? ______________________

____

<u>Almost finished. Now some questions about barriers to the prescription of PrEP...</u>

How relevant are the following barriers for potential PrEP users according to your personal experience? *(items presented in randomised order)*

	No relevance – 0	*1*	*2*	*3*	*4*	*5*	*6*	*7*	*8*	*9*	*Highest relevance – 10*
Worries about mild or temporary side effects	O	O	O	O	O	O	O	O	O	O	O
Worries about severe or permanent side effects	O	O	O	O	O	O	O	O	O	O	O
Worries about getting infected with other STIs	O	O	O	O	O	O	O	O	O	O	O
The monthly costs for the PrEP medication	O	O	O	O	O	O	O	O	O	O	O
The costs for the laboratory investigations	O	O	O	O	O	O	O	O	O	O	O
Cultural barriers	O	O	O	O	O	O	O	O	O	O	O
Assessment of their own risk of getting infected with HIV as too low to take PrEP	O	O	O	O	O	O	O	O	O	O	O

Time required for regular visits to the doctor	O	O	O	O	O	O	O	O	O	O	O
Difficulties finding a doctor who prescribes PrEP	O	O	O	O	O	O	O	O	O	O	O
Worries about stigmatization in the peer group	O	O	O	O	O	O	O	O	O	O	O
Lack of information about PrEP in client-friendly language	O	O	O	O	O	O	O	O	O	O	O
Lack of information about PrEP in the native language of the client	O	O	O	O	O	O	O	O	O	O	O
The time required for PrEP management is too high for the doctors											
It is difficult for doctors to identify PrEP candidates											

Are there any other relevant problems for potential PrEP users that occur in your personal counseling practice?
Please specify these here... ____________________________________

<u>Two final questions:</u>

Which of the following information or training materials would improve counselling on PrEP or make it more practical? *(Multiple answers allowed)*

O A clinical practice guideline that provides a good overview of indications, contraindications and necessary laboratory investigations
O A decision-aid for clients that provides information on PrEP in patient-friendly language
O A decision-aid for clients that provides information on PrEP in different languages
O An app- or SMS-based reminder for PrEP users to promote adherence
O Information and training for physicians on the management of PrEP (e.g. medical investigations)
O Information and training for physicians on the identification of PrEP candidates
O Information or training on “Talking with patients about sexuality”
O Other: ____________

Are there other aspects or problems regarding PrEP that you note? Do you have any other suggestions on how to improve counselling and PrEP implementation among persons who may benefit from PrEP?
Please specify these here ... __________________

Thank you for participating in the survey!
